# The Hexaco Personality Traits of Higher Achievers at the University Level

**DOI:** 10.3389/fpsyg.2022.881491

**Published:** 2022-04-15

**Authors:** Ruofan Jia, Rabia Bahoo, Zhendong Cai, Musarrat Jahan

**Affiliations:** ^1^School of Humanities and Social Science, Xi’an Jiaotong University, Xi’an, China; ^2^Department of Education, The Government Sadiq College Women University Bahawalpur, Bahawalpur, Pakistan; ^3^Department of Philosophy and Religion, Research Center for Philosophy of Science and Technology, Northeastern University, Shenyang, China; ^4^Department of Special Education, The Islamia University of Bahawalpur, Bahawalpur, Pakistan

**Keywords:** personality traits, Hexaco model of personality, emotionality, honesty, openness to experience, extraversion, agreeableness, conscientiousness

## Abstract

This study attempted to explore the personality traits of higher achievers at the university level. The core objective of this investigation was to illustrate the nature of personality traits of the higher achievers’ students. To study this phenomenon, a quantitative research approach was used. The students were chosen by using a purposive sampling technique and included 758 high achievers enrolled in various programs at the Chinese universities. Based on the Hexaco model of personality, a questionnaire was used to gather information from respondents as a research tool to examine the personality traits of position holders after an extensive review of the relevant literature. Tool validity was determined by following the face, content, construct (convergent and discriminant validity) validation process. This investigation concluded that honesty, emotionality, and openness to experience were very high among the higher achievers’ students. Only honesty in female higher achievers’ students was significantly high than male, remaining factors “extraversion, agreeableness, conscientiousness, and openness to experience” were significantly high among male higher achievers’ students. Moreover, the higher achievers of science group students were more extraversion, agreeableness, and conscientiousness than arts group students. However, higher achievers in hostels were more emotional and agreeableness than the day scholars. Overall step-wise regression analysis, indicated that agreeableness and extraversion factor has significant influence on higher achievers.

## Introduction

All human beings are born different. They have their own preferences to live a life and choose to carry out their work. Despite their diversity, people share some common traits and behaviors, such as modesty, sincerity, fairness, social boldness, forgiveness, liveliness, gentleness, Conscientiousness and inquisitiveness, when they join the world. Likewise, they have their own capacities to understand the world, people, and happenings. As a result, the beauty of this planet is that every single person has their own distinct behavior and persona (Geramian et al., 2012). Due to their individuality and personality, they show their interests, likes, dislikes, feelings, and give diverse opinions. Thus, all human beings, although being equal but carry their personalities so differently (Goldberg, 1992).

Globalization, technology, and informational developments have posed difficulties to the universities throughout the world in the 21st century. As a result, universities must equip their students with new skills, information, and competencies in order to cope with new challenging tasks that are in accordance with national or international educational aims and standards in order to remain competitive and relevant. Students, on the other hand, have individual perspectives and diverse qualities that cause them to interpret world views differently and act differently in different educational milieus.

Eulaica (2020) mentioned that “Innate cognitive ability is a key predictor to academic success.” The idea of inborn cognitive talent is acceptable in several fields of the educational settings. Furthermore, a recent study has demonstrated that non-cognitive traits are equally important for attaining high-academic performance (Ciorbea and Pasarica, 2013). Personality is one among the key factors of academic performance which has non-cognitive characteristics for learning (Eulaica, 2020). Non-cognitive factors such as personality characteristics have been identified in the literature as predictors of learning performance.

Therefore, exploration of those factors which affect academic achievements is one of the focal points in the research field of psychology because of its noteworthy implications for both learning and its pedagogy (Zeb et al., 2021). Generally, it is observed that at a higher education level learning is task-oriented as students are perceived more self-directing and self-regulating, as these qualities of adult learners’ demand self-diagnosing needs so it is necessary for the instructor to consider diverse personality traits while planning learning tasks (Baiocco et al., 2017). As a result, instructors can benefit from taking these disparities into consideration when assessing the unique distinctions among their adult pupils (Stroh et al., 2005; Nordin et al., 2020). Personality is a broad term that generally incorporates all of these changes. In general, the key contributory variables for the formation of a student’s personality are family, peer group, media, educational institution learning environment, etc. (Alberts, 2010; Hansen, 2011). Hence, personality has a great influence on what an individual thinks, their opinions, verdicts, capabilities, and necessities. A person’s judgment about other individuals is based upon their personality (Ahmed, 2017). Furthermore, in view of Sulaiman (2019), during teaching and learning processes, it is necessary to deal with socially and psychologically disturbed children in the classroom. If pupils are unable to perform adequately, effective teaching has not occurred.

## Literature Review

### Concept of Personality and Personality Traits

Philosophers, psychologists defined, explained, and explored personality and personality traits in their own thoughts and judgments. Most of the theories go beyond the basic definition of personality and they cannot effectively give the literal meaning of personality. The entomology of the word personality is taken from the two Greek words “*Per*” and “*Sonare.*” Later on, this word was changed to “Persona.” The word persona is a noun and a name of the mask which actors wear on the theater to show their personalities in different characters. It was used in ancient Greek to entertain the audience by the mask in order to perform well and to portray the real picture of the performed character to the audience. This word reflects the personality of the person in two senses that the person performs in the life which character he or she has been given and the second the person could behave which he or she is not in actual sense (Shian et al., 2022). American Psychology Association highlighted with reference to Encyclopedia of psychology that “The study of personality focuses on two broad areas: One is understanding individual differences in particular personality characteristics, such as sociability or irritability. The other is understanding how the various parts of a person come together as a whole” (American Psychological Association, 2020, para 1).

In short, personality assessment is a very challenging task in order to understand its every aspect effectively and logically. The personality can be categorized into two major types one is optimistic and the other one is pessimistic. The individual having an optimistic personality, thinks positive and always hopes well while contrastingly the individual having pessimistic personality traits is always superconscious and afraid of taking challenges. As indicated by Warr (1999), personality is a permanent trait of a person that indicates long-term and persistent individual distinctions in emotive style and has a similar influence on the visceral outburst. Several studies (McAdams and Pals, 2006; Fleeson and Gallagher, 2009) noted that personality characteristics are defined as the distinctions in an individual’s frequency and intensity of thinking, behaving, and feeling in certain ways. Whereas McCrae and Costa (1999) and Zillig et al. (2006), defined personality traits are characterized as a person’s generally constant patterns of behavior, motivation, emotion, and cognition.

Personality, according to psychologists, refers to one’s style of thinking, performing, and experiencing. Consistent and distinctive manners and styles of thinking, feelings, and activities are presented in an appropriate sequence in the case of peculiarities. When we talk about personality, we assume the full picture of something or someone. Regarding this perspective, personality is defined as a person’s constant and consistent attitude in all circumstances.

Therefore, diverse facets of human personality play a role in its development. Dominance of one or more than one element gives a distinctive shape to the personality. Features of human personality such as sincerity, modesty, social boldness, forgiveness, and humanity, are some major components of human personality that affect their internal life (Abu-Raiya, 2014). Hence, personality traits are important factors to understand the behavioral aspects of one’s personality. These personality traits are basically categorized into two types which are mean-level and individual-level traits. These basic traits are further subdivided into further subcategories which are: honesty–humility, extraversion agreeableness, consciousness, emotional stability, and openness to experiences. As the aforementioned traits, each of the traits has its own individuality and descriptive value. The major focus of this research work is to understand the personality of higher achievers by these traits.

The Big Five-Factor model, which refers to “extroversion, openness to experience, agreeableness, conscientiousness, and neuroticism,” is one of the most extensively used assessment techniques for assessing personality traits (Weisberg et al., 2011; Mata et al., 2021). This study employed the Hexaco personality model, which is an extension of the Big Five Factor Model. The traits of the Hexaco model have similarities with other dimensions of personality models. Although, this model is quite different due to the addition of the H factor, i.e., honesty–humility. Six factors of personality of the Hexaco model were identified and calculated with the help of questions. These questions were designed to measure the individual’s personality. Ashton and Lee designed a self-based and observation-based inventor to analyze personality traits. This model was used to describe with detail of six major components of human personality. The Hexaco model of personality (six factors) with their adjective’s justifications specified later (Ashton and Lee, 2007, 2010; Abbasi et al., 2020).

#### Honesty–Humility

Individuals who scored high on the honesty and humility scale are expected to be honest in their interactions. These individuals never take advantage of others for their personal gain. They adhere to the laws and are uninterested in a lavish lifestyle. They do not expect any pretentious or social status from anyone. Contrastingly, persons who are less responsive to honesty and humility, they are more dishonest in public dealings. They feel at ease taking advantage of others for their personal gain. They have the ability to effortlessly breach the law for their personal gain. They are capitalistic in their outlook. These types of people are entirely concerned with themselves at all times throughout their lives (Ashton and Lee, 2009; Camps et al., 2016).

#### Emotionality

According to Ashton and Lee (2009), emotionality is characterized by fearfulness/worriedness, mushiness, nervousness, and helplessness. An emotional person is expected to be emotional by nature and he or she place a high value on emotions in any relationship, they are quite sensitive in their daily activities and need sympathy from others (Othman et al., 2020). Moreover, they express anxiousness when they encounter some negative experience. Individuals with low emotionality are less sensitive, they do not require emotional attachment from others, and have a more relaxed attitude while under stress (Camps et al., 2016; Zeb et al., 2021).

#### Extraversion

Extraversion is considered the positive nature of persons which falls in the category of social self-esteem, self-confidence, social-audaciousness, seeking of excitement, positive emotions, sociability, and liveliness (McCrae and John, 1992). Individual differences in social interactions, assertiveness, and energy level are referred to as extraversion personality traits. A person who is extraverted, enjoys social gathering and enjoys confidently every event of their life (Ashton and Lee, 2009). These people are energetic and can face every challenge of life bravely, and they experience positive emotions such as enthusiasm and excitement. Introverts, on the other hand, are socially and emotionally repressed and conservative (Hakimi et al., 2011). People who do not pose any characteristics in this factor of personality they remained unsocial. They cannot feel relaxed in a social jamboree or gatherings. Hence, such kinds of people are pessimistic by nature and love to live in their own world because they do not want to become the center of attraction (Ashton and Lee, 2009).

#### Agreeableness (vs. Anger)

Agreeableness is characterized by forgiveness, gentleness, low self-confidence, flexibility morality, high levels of trust in others, and patience. Such types of individuals have the ability to forgive others for their errors. Their nature is characterized by flexibility. They judge others with sympathy and are willing to work with others because of their adaptable nature. In short, individuals who are agreeable have empathetic care for the well-being of others, treat everyone fairly and with respect, they usually have good views about others (Ashton and Lee, 2009). Disagreeable people have low regard for others. Because such kind of pupils show a fiery temperament when confronted with serious wrongdoing by others. They pass judgment on others and do not allow for flexibility. They have a short temper, therefore, if somebody misbehaves, they will react aggressively.

#### Conscientiousness

Competence, continuous effort, self-discipline, organization, goal orientation, and striving for accomplishment are all traits of conscientiousness (McCrae and Costa, 1990), with a high degree of deliberation allowing conscientious persons to evaluate the pros and cons of a particular circumstance (Johnson, 1997). Othman et al. (2020) denoted that in conscientiousness “an individual is reliable, cautious, competent, accountable, prepared, hardworking, and productive.” It also refers to tenacity, determination, and performance in the profession as well as in the area of teaching and learning (McCrae and Costa, 1999). These pupils are more self-disciplined. As they are extremely conscientious in the pursuit of their goals, therefore, they prefer to complete their tasks on time and follow established guidelines. Moreover, they are perfectionists and never make hasty decisions likewise; they are proficient in comprehending any new situation and make valuable decisions about the situation. Students with poor conscientiousness are unable to tackle any problem with confidence. They won’t be able to attain their objectives because they have low self-esteem to deal with every new situation as it arises. They feel satisfaction even with a work of less importance (Ashton and Lee, 2009, 2010).

#### Openness to Experience

Hakimi et al. (2011) concluded that openness to experience “reflects an individual’s broad-mindedness, depth of attitude, and penetrable awareness.” Openness to experience is reflected in a person who is creative, imaginative, and curious as opposed to concrete-minded and narrow thinking (McCrae and John, 1992). A person of aesthetic nature is someone who can respond honestly to any event. He is more sensitive to the beauty of nature than other members of society. He is a man who is always on the lookout for new information in all the areas of life. These people have a creative mind full of imagination and are always thinking about new things. The less flexible people, openness to experiences is less artistic by nature. They are less interested in innovative activities and want to live a quiet and simple lifestyle. These people’s perspectives are devoid of creativity and novelty.

The development of positive traits is the highway to an individual’s aims, whatever they may be. A tiny modification in personality may make a tremendous impact on the goal-setting and organization of one’s life. In short, an individual’s success is determined by his personal qualities. Therefore, the purpose of this study was to look into the personality qualities of universities’ position holders, which will assist teachers to understand what sorts of personality traits higher achievers have so that they may better educate students and address the difficulties of the higher achievers.

Numerous studies on high achievers have been published on various variables. Research on study habits and academic achievement was presented by Kapoor (1987), Yip (2007), and Haider et al. (2021). A study on academic achievement and self-concept was conducted by Singh (1983) and Adsul (2011). Study on socio-economic status and academic achievement were explored in the several studies (Nair, 1987; Trivedi, 1988; Ganguly, 1989; Singh, 1989; Davanesan, 1990; Mohanty, 1992). Creative thinking abilities and academic achievement (Mishra, 1978; Anwar et al., 2012).

## Materials and Methods

### Objectives of the Study

The prime objectives of this current investigation are as follows:

1. To depict the nature of personality traits of higher achievers’ students at the university level.
2. To compare the higher achievers’ personality traits of male and female students.
3. To compare the higher achievers’ personality traits of science and arts group students.
4. To compare the higher achievers’ personality traits of hostilities and day scholars.
5. To investigate the effect of the Hexaco model of personality traits on higher achievers’.

### Significance of Investigation

Exploring differential dimensions of the Hexaco model of personality traits on higher achievers will not only be a significant addition to current knowledge but will also give a theoretical background to educationists, psychologists, and educational psychologists. This research will be helpful for teachers to see what types of personality traits position holders have so that they should teach students in a better way and solve their educational problems accordingly and assist the low-grade student how they flourish and groom their personality.

University graduates are vital in forming the future of the country. Usually, students at elementary, secondary, and higher secondary levels are in the phase of personality grooming and they did not have permanent personality traits as during this stage of personality development many changes possibly occur so according to this perspective, the researcher approached the respondents at the university level because at this stage of education majority of students’ personality has been shaped and students are about to start their professional life. As mentioned by Umar et al. (2010), the reflective thinking process starts at late adolescence and early adulthood so at this stage they have several opportunities to interact with diverse peers, relatives, and even sometimes with their teachers. Minds are broadened and personalities are groomed up at this age to take personal or professional grits, willpowers, and decisions. The efforts of higher education graduates are next to be put in the field/practical life therefore university students were the prime focus of this study. Furthermore, the research investigation was intended to observe personal traits in diverse cultures so, the Chinese universities were selected as the population of the study. Further, in this regard, the results of the study would more practical and reliable.

### Methodology

This research is conducted at the university level, and it was designed to measure the Hexaco model of personality traits of the higher achievers’ students enrolled at different universities in different programs. This research was descriptive in its nature as the research deals with an existing situation. A survey method was adopted to collect data. Cohen et al. (2007) stated that descriptive research is an appropriate approach to study an existing situation. The researcher conducted this investigation in a quantitative form since the data are best presented in terms of mean scores, SD, the independent *t*-test and step-wise regression analysis The calculated data interpreted as findings in the light of the study objectives.

### Population and Sample Size and Technique

The population was the entirety of the observation made on all the objects having some common talents, abilities, and a set of qualities, which were the specific interest to research. “Targeted population” of the study was consisted of all male and female higher achievers’ students enrolled in different programs in the public and private universities of all over China. The purposive sampling technique was used to select the students and was consisted of 758 high achievers.

### Development of Research Tool and Its Validation Process

For this study, relevant literature was reviewed and a questionnaire was developed based on the Hexaco model of personality traits. It was kept in view that each statement must express a definite idea. The questionnaire was on five-point Likert scale containing 49 items out of which 38 were selected. The questionnaire was comprised of two sections: the first section was comprised of demographic variables such as institution, program, semester, department, CGPA, and position in class. Furthermore, the second section included 38 items (six factors) about personality traits. The validity and estimated reliability of the questionnaire is listed later.

### Convergent Validity

Convergent validity (CV), as defined by Urbach and Ahlemann (2010), is the degree to which indicators that indicate a concept converge in respect to items measuring other constructs. This CV is assessed by two measures, the first is known as average variance extracted (AVE), and the second one is known as item inter reliability or FL values. This was proposed by Fornell and Larcker (1981). If the values of the AVE construct are greater than 0.5, then the construct has an adequate convergent validity, and if the FL values are greater than 0.6, the construct has also adequate convergent validity. The FL and AVE values are shown in Table 1.

**TABLE 1:** Measurement model.

Factor loading		Average variance extracted values (AVE)	Cronbach’s alpha	Composite reliability
Honesty-Humility		0.639	0.771	0.778
H-1	0.73			
H-2	0.67			
H-3	0.69			
H-4	0.90			
H-5	0.76			
H-6	0.71			
H-7	0.81			
Emotionality		0.649	0.756	0.844
E-1	0.76			
E-2	0.75			
E-3	0.78			
E-4	0.77			
E-5	0.75			
E-6	0.79			
Extraversion		0.575	0.732	0.851
E-1	0.69			
E-2	0.74			
E-3	0.76			
E-4	0.73			
E-5	0.77			
E-6	0.67			
E-7	0.70			
Agreeableness		0.793	0.763	0.884
A-1	0.74			
A-2	0.96			
A-3	0.76			
A-4	0.80			
A-5	0.81			
A-6	0.87			
Conscientiousness		0.603	0.807	0.862
C-1	0.79			
C-2	0.69			
C-3	0.72			
C-4	0.75			
C-5	0.77			
C-6	0.85			
Openness to experience		0.522	0.771	0.845
O-1	0.86			
O-2	0.69			
O-3	0.70			
O-4	0.91			
O-5	0.88			
O-6	0.75			

The aforementioned table displays that all factors of the Hexaco model of personality traits remain greater than the 0.6 value that exemplifies an acceptable range. The factor loading values of the honesty–humility (0.67–0.90), emotionality (0.75–0.79), extrovert (0.69–0.77), agreeableness (0.74–0.96), conscientiousness (0.69–0.85), and openness to experience (0.69–0.91). In Table 1, the second column reveals that the AVE values range from 0.522 to 0.793, these values of AVE for the constructs are greater than the minimum allowed value of 0.50, which shows acceptable convergent validity. The highest value of AVE is found in emotionality (0.649) and the lowest value is found in openness to experience (0.522). Internal consistency reliability (ICR) is normally determined by the Cronbach’s Alpha and through composite reliability (CR) analysis. Nunnally and Bernstein (1994) reported that for exploratory research, the adequate values for Cronbach’s alpha and CR must be above than 0.7 and values above 0.8 are desirable for the confirmatory research. However, values less than 0.6, point out a lack of internal consistency. As depicted in Table 1, third and fourth columns present the values regarding Cronbach’s alpha and CR, respectively. In all dimensions, Cronbach’s alpha and CR values are substantially above the suggested level of 0.70.

### Discriminant Validity

Urbach and Ahlemann (2010) mentioned that discriminant validity assesses how much the signs of latent variables (LVs) are likewise unique in relation to one another. Discriminant validity determines whether or not a construct indicator is simultaneously measuring another construct. The Fornell-Larcker (FL) criteria and cross-loadings (CLs) criteria are used in the PLS–SEM technique to assess discriminant validity (Fornell and Larcker, 1981). By comparing the FL and CL of all the signs to their corresponding LVs, the discriminant validity of the measurement model can be evaluated. To attain CLs, each construct’s score is connected with all other indicators (Chin, 1998). When an indicator’s loading values are greater in contrast to its own measured construct than against any other construct, and each construct has the highest values with its assigned indicator, discriminant validity may be confirmed and inferred. Table 2 clarifies the Fornell–Larcker criterion used in this study’s model. The values in the table are higher than the values in their respective column and row, as shown in Table 2. This demonstrates that discriminant validity is adequate.

**TABLE 2:** Fornell–Larcker criterion.

Factors	1	2	3	4	5	6
1. Honesty-Humility	0.734					
2. Emotionality	0.233	0.741				
3. Extraversion	0.277	0.511	0.758			
4. Agreeableness	0.142	0.256	0.186	0.89		
5. Conscientiousness	0.137	0.211	0.311	0.547	0.714	
6. Openness to experience	0.339	0.511	0.477	0.214	0.208	0.723

### Data Collection and Data Analysis Procedure

This study examined the personality traits of higher achievers’ students. The survey was carried out personally during the months of December 2020. After collecting the data, it was scrutinized to observe the personality traits of position holders and the results were analyzed by using SPSS “Statistical Package for the Social Sciences” software version 22. To attain the results of objective 1 descriptive analysis was applied in order to see the nature of personality traits of the higher achievers’ students based on the Hexaco model of personality. Objectives 2, 3, and 4 were assessed through the analysis of an independent *t*-test. Objective 5, was assessed through stepwise regression analysis.

### Demographics of Study Respondents

Table 3 revealed the demographic information of the study participants. The sample data reveals that the total number of respondents was 758 out of which 402 (53%) were male and 356 (47%) were female. There were 519 (68.5%) respondents, who belonged to day scholars, while (239) 31% were hostelites. Furthermore, sample data depict those 446 (59%) respondents were studying science subjects, and 312 (41%) were from Arts subjects.

**TABLE 3:** Demographics of study respondents.

Demographics	Frequency	Percentage
**Gender**		
Male	402	53
Female	356	47
**Residential**		
Day-scholar	519	68.5
Hostelites	239	31.5
**Group**		
Science	446	59
Arts	312	41

#### Research Objective 1: To Depict the Nature of Personality Traits of Higher Achievers’ Students at the University Level

In Table 4, the result of this study depicts the nature of higher achievers’ students’ personality traits at the university level. The mean scores were classified in descending way from 4.28 to 3.74, indicating that these scores were between high to low. This investigation evident that the position holders felt that, they were more inclined to display high personality traits of openness to experience. The mean score of openness to experience was remained (*m* = 4.38, SD = 0.563), emotionality (*m* = 4.04, SD = 0.608), and honesty (*m* = 4.02, SD = 0.603). The study result also showed that these position holders students also displayed moderate personality of extraversion (*m* = 3.80, SD = 1.03), conscientiousness (*m* = 3.79, SD = 0.601), agreeableness (*m* = 3.78, SD = 0.905). Captivatingly, the results of extraversion, conscientiousness, and agreeableness were also in the acceptable range.

**TABLE 4:** Personality traits of higher achievers’ students at the university level.

Factors	Mean	Std. deviation
Honesty-Humility	4.02	0.603
Emotionality	4.04	0.608
Extraversion	3.80	1.03
Agreeableness	3.78	0.905
Conscientiousness	3.79	0.601
Openness to experience	4.38	0.563

#### Research Objective 2: To Compare the Personality Traits of Male and Female Students

Table 5 depicts the difference between personality traits of male and female students. Mean value shows that the honesty in female students was significantly high than male (*m* = 4.08, *t* = −3.295, *P* = 0.001). Emotionality mean value indicates that female (*m* = 4.11, *t* = −1.629, *P* = 0.103) students were more emotional than male students but this difference remained insignificant. Extraversion (*m* = 3.90, *t* = 5.342, *P* = 0.000), agreeableness (*m* = 3.80, *t* = 3.089, *P* = 0.002), conscientiousness (*m* = 3.80, *t* = 3.190, *P* = 0.001), and openness to experience (*m* = 4.35, *t* = 4.23, *P* = 0.000) were significantly different and high among male students. The *t*-value indicates that extraversion in male students was high than other factors.

**TABLE 5:** Comparison between male and female higher achievers’ students’ personality traits.

Factors	Gender	Mean	Std. deviation	*t*-value	*P*
Honesty-Humility	Male	4.00	0.633	−3.295	0.001
	Female	4.08	0.609		
Emotionality	Male	4.07	0.631	−1.629	0.103
	Female	4.11	0.626		
Extraversion	Male	3.90	0.955	5.342	0.000
	Female	3.68	0.977		
Agreeableness	Male	3.80	0.876	3.089	0.002
	Female	3.69	0.953		
Conscientiousness	Male	3.80	0.750	3.190	0.001
	Female	3.70	0.799		
Openness to experience	Male	4.35	0.767	4.234	0.000
	Female	4.20	0.919		

#### Research Objective 3: To Compare the Personality Traits of Science and Arts Students

Table 6 shows the difference between personality traits of science and arts group students. Mean values show that honesty (*m* = 4.11, *t* = −3.216, *P* = 0.001), extraversion (*m* = 3.84, *t* = 3.99, *P* = 0.000), agreeableness (*m* = 3.84, *t* = 8.750, *P* = 0.000), and conscientiousness (*m* = 3.82, *t* = 2.131, *P* = 0.033), were significantly different in science and arts group students. Moreover, the negative *t*-value indicates that arts student were more honest than science students while positive *t*-values indicated that science student were more extraversion, agreeableness, and conscientiousness than arts students.

**TABLE 6:** Comparison between science and arts higher achievers’ students’ personality traits.

Factors	Group	Mean	Std. deviation	*t*-value	*P*
Honesty-Humility	Science group	4.01	0.60	−3.216	0.001
	Arts group	4.11	0.68		
Emotionality	Science group	4.09	0.62	0.821	0.411
	Arts group	4.07	0.64		
Extraversion	Science group	3.84	1.02	3.995	0.000
	Arts group	3.65	1.12		
Agreeableness	Science group	3.84	1.02	8.750	0.000
	Arts group	3.65	1.12		
Conscientiousness	Science group	3.82	0.84	2.131	0.033
	Arts group	3.46	1.09		
Openness to experience	Science group	3.77	0.75	1.216	0.224
	Arts group	3.69	0.83		

#### Research Objective 4: To Compare the Personality Traits of Hostelites and Day’s Scholar

Table 7 illustrates the difference between personality traits of hostelites and day scholars. Mean values spectacle that only emotionality (*m* = 4.12, *t* = −2.012, *P* = 0.044), and agreeableness (*m* = 3.80, *t* = −2.315, *P* = 0.021), were significantly different in hostelites and day scholars. Moreover, the negative *t*-value indicates that hostelites student were more emotional and agreeableness.

**TABLE 7:** Comparison between hostelites and day’s scholar higher achievers’ students’ personality traits.

Factors		Mean	Std. deviation	*t*-value	*P*
Honesty-Humility	Day scholars	4.04	0.63	0.565	0.572
	Hostelites	4.03	0.60		
Emotionality	Day scholars	4.07	0.63	−2.012	0.044
	Hostelites	4.12	0.61		
Extraversion	Day scholars	3.78	1.05	−0.957	0.339
	Hostelites	3.82	1.05		
Agreeableness	Day scholars	3.72	0.95	−2.315	0.021
	Hostelites	3.80	0.81		
Conscientiousness	Day scholars	3.75	0.77	0.090	0.928
	Hostelites	3.75	0.76		
Openness to experience	Day scholars	4.29	0.91	1.133	0.257
	Hostelites	4.25	0.90		

#### Research Objective 5: To Investigate the Effect of the Hexaco Model of Personality Traits on Higher Achievers’

Table 8 elaborates stepwise multiple regression. In step one, the study analysis discloses that agreeableness separately, has a significantly negative influence (*R*^2^ = 0.003, *b* = −0.052) on higher achievers, however, adding of agreeableness and extraversion in step 2 among higher achievers. This investigates that in step two agreeableness has significantly negative influence (*R*^2^ = 0.006, *b* = −0.071) but extraversion has significantly positive influence (*b* = 0.062) on higher achievers. This study findings display that all out of six factors of the Hexaco model of personality traits, only two factors are significant.

**TABLE 8:** Stepwise multiple regression to find out the effects of personality traits on higher achievers’ students.

Model		Unstandardized coefficients beta (Std. error)	Standardized coefficients beta	*t*	Sig.	*F*	*R*	*R* ^2^
	1					7.453	0.052	0.003
(Constant) Agreeableness		8.109 (0.307)		26.419	0.000			
		−0.217 (0.080)	−0.052	−2.730	0.006			
	2					8.671	0.079	0.006
(Constant) Agreeableness extraversion		7.540 (0.356)		21.184	0.000			
		−296 (0.083)	−0.071	−3.551	0.000			
		0.227 (0.072)	0.062	3.141	0.000			

*^a^Predictors: (Constant), Agreeableness;*

*^b^Predictors: (Constant), Agreeableness, Extraversion.*

## Discussion and Conclusion

The current investigation attempted to explore the personality traits of higher achievers at the university level, taking into account gender, residential status (Day scholars and Hostelites), and groups (Science and Arts) exposure differences. The results of this study depict the nature of the higher achievers’ students’ personality traits in the universities of China. This investigation evident that, the higher achievers’ students display high-personality traits of openness to experience, emotionality, and honesty while they display a moderate level of personality in terms of extraversion, conscientiousness, and agreeableness. The difference between personality traits of male and female students was also found. The honesty in female higher achievers’ students was significantly high than male students, while four factors regarding the Hexaco model of personality traits (extraversion, agreeableness, conscientiousness, and openness to experience) were significantly high among the male higher achievers’ students.

On the other hands, study results also illustrate that honesty, extraversion, agreeableness, and conscientiousness were significantly different in the science and arts group students. Science higher achievers’ students were more extrovert, flexible, and perfectionist than arts higher achievers’ students. The results of emotionality and agreeableness factors were significantly different in hostelites and day scholars’ students. The results indicate that hostelites students were more emotional and gentler. Step-wise regression analysis explained that agreeableness and extraversion factor has a significant influence on higher achievers. Likewise, extraversion has a positive influence on higher achievers whereas, agreeableness remained negative. The existing research is one of the first studies which examine and explore the personality traits of higher achievers at the university level based on the Hexaco model of personality. To the best of our knowledge, we could not find any research paper regarding this investigation but various research was found on various aspects such as personality traits relationship, effect, impact, and influence on the academic achievements or learning outcomes and these researchers were merely in a descriptive manner. Our study offered a more comprehensive view of the phenomenon by investigating different types of traits in a more extensive sample. Moreover, it is also worth to note that Day’s scholar and hostelites, science and arts higher achievers’ orientation differences in personality have never been investigated in the literature.

### Theoretical and Practical Implication

The research study adds in the existing body of knowledge on factors related to the students’ high achievement scores in academics. This study highlights the personality attributes of high achievers which enable the effective work with students having such qualities and characteristics. By promoting such instructional activities which are related to certain personality attributes of their students in the light of this study’s findings. Hence, it gives a new direction to the future researchers too by exploring new dimensions of existing theory not only on higher achievers but also on low achievers. In practical settings, the research will facilitate the university teachers to produce more high achievers and also assist students to groom their personality traits to become high achievers (Baiocco et al., 2017). The study will empower the low achievers by giving them a direction to overcome their negative and toxic personality traits. Thus, the study will help the university students of our country to be more productive to improve their overall performance. Furthermore, social experiences and behaviors are uncontrollable and unpredictable for institutions, the universities must conduct observational assessments of undergraduates at each semester’s completion and employ counselors to minimize their negative personality behaviors. Hence, state, teachers, and parents would be able to conduct activities accordingly to shape and nourish good personality traits. The management of the university should give awareness to the teachers on how they can play their role by developing strategies for the grooming of students’ personalities.

### Limitation and Future Research

The findings of this study should be seen in the light of a number of caveats. First, this study was conducted in a sample of university students, and it was conducted in a Chinese cultural setting, exclusively with the Chinese students, and at a unique time (during the pandemic), limiting the feasibility of generalizing the conclusions to some extent. The study data were gathered from postgraduate students from two different disciplinary areas (science and arts). However, the students from other disciplines were not included in the sample, and future researchers should focus on them. Moreover, a longitudinal study ought to be carried out through primary to elementary and secondary school levels, so that long-term results of prior processes can also be reported. Furthermore, future study efforts may focus on this topic through observation or interviews. Moreover, there is no study found on the perspired population on the personality traits of higher achievers, so there is a lack of relevant literature to confirm the findings of the current research study. As this research only investigates the personality traits of higher achievers, new researchers can also observe the personality traits of low achievers. Further comparative researches can also be conducted to compare the personality traits of higher achievers with low achievers. Researchers can also examine the impact of general and cultural aspects of multi-ethnic societies on the personality traits of students. As this study was a survey based on a quantitative approach which was based on the determinants that were research-based only. However, to explore further prospects of personality qualitative studies are recommended.

## Data Availability Statement

The raw data supporting the conclusions of this article will be made available by the authors, without undue reservation.

## Author Contributions

All authors listed have made a substantial, direct, and intellectual contribution to the work, and approved it for publication.

## Conflict of Interest

The authors declare that the research was conducted in the absence of any commercial or financial relationships that could be construed as a potential conflict of interest.

## Publisher’s Note

All claims expressed in this article are solely those of the authors and do not necessarily represent those of their affiliated organizations, or those of the publisher, the editors and the reviewers. Any product that may be evaluated in this article, or claim that may be made by its manufacturer, is not guaranteed or endorsed by the publisher.
